# Maturational Changes in Action‐Effect Integration Processes Are Reflected by Changes in the Directed Cortical Network Communication

**DOI:** 10.1002/hbm.70339

**Published:** 2025-09-08

**Authors:** Jasmin Mayer, Moritz Mückschel, Bernhard Hommel, Christian Beste

**Affiliations:** ^1^ Cognitive Neurophysiology, Department of Child and Adolescent Psychiatry, Faculty of Medicine TU Dresden Dresden Germany; ^2^ School of Psychology Shandong Normal University Jinan China; ^3^ German Center for Child and Adolescent Health (DZKJ), Partner Site Leipzig/Dresden Dresden Germany

**Keywords:** adolescence, adults, directed connectivity, neural maturation, theta oscillationsaction effect integration

## Abstract

Acting intentionally is a major aspect of human cognitive development and depends on the ability to link actions with their consequences. Action‐effect binding (AEB) is a fundamental mechanism enabling this. While AEB has been well‐characterized in adults, its neurophysiological underpinnings during adolescence remain unclear. This study investigates differences between adolescence and adulthood in the directed cortical network communication underlying AEB. Using an EEG frequency tagging approach, we examined differences in theta‐driven directed connectivity between adolescents and adults. Our findings reveal that both groups engage a core network comprising the insular cortex, anterior temporal lobe, and inferior frontal cortex. However, adolescents exhibit stronger directed connectivity within this network, particularly in anterior temporal lobe‐mediated interactions, suggesting a greater reliance on representational processing for action‐effect integration. Furthermore, adolescents uniquely recruit posterior ventral stream regions, including the lingual gyrus. This additional involvement suggests an increased demand for sensory integration in adolescents, potentially compensating for immaturities in action‐effect representation. These results indicate that while the essential neural architecture for AEB is established in adolescence, its functional organization differs from that of adults. This study provides novel insights into developmental changes in cortical network communication underlying intentional action control.

## Graphical abstract

The work shows how different directed communication patterns (i.e., information transfer) between cortical regions serve intentional behavior in different stages of human development.

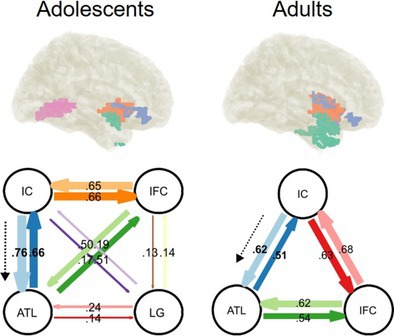

## 1 Introduction

Acquiring the ability to act intentionally is a major achievement of human cognitive development (Vygotskiĭ et al. 2012; Zelazo 2004). Cognitive science has proposed that the ability to link actions with their consequences is a foundational aspect of human cognition, enabling individuals to engage in goal‐directed behaviors effectively (Eenshuistra et al. 2004). This process, known as action‐effect binding (AEB), allows the brain to anticipate the outcomes of actions and to plan future actions based on these expectations (Hommel et al. 2001). The establishment of action‐effect associations is underpinned by intricate neural mechanisms that involve the integration of sensory and motor information into what is known as event files—representations of sensory and motor aspects of an action and its environmental effect that guide future behavior (Hommel et al. 2001; Hommel 2004). Once formed, AEBs can be reactivated through internal representations, anticipation, or external stimuli (Elsner and Hommel 2001; Hommel 2022; Kühn et al. 2011).

Executing an intentional action involves activating the neural representation of the intended action‐effect, triggering the associated AEB and spreading activation to the corresponding movement pattern (Hommel et al. 2001; Shin et al. 2010; Waszak et al. 2012). The action's success is evaluated by comparing actual sensory effects with expected outcomes (Waszak et al. 2012). This is in line with ideomotor theories of action control, which propose that actions are coded in terms of their intended goals (Elsner and Hommel 2001), emphasizing the role of anticipating action‐effects in voluntary action control.

While AEB mechanisms are already present from infancy (Eenshuistra et al. 2004; Verschoor et al. 2010), their efficiency and application develop considerably with age. Younger individuals, particularly children and adolescents, show slower acquisition and weaker retention of action‐effect associations (Karbach et al. 2011; Verschoor et al. 2013), which can hinder their ability to use these associations for intentional action selection. Despite consistent findings of temporal binding—the subjective compression of time between actions and outcomes—across age groups (Lorimer et al. 2020), the ability to leverage AEBs for goal‐directed control becomes more sophisticated with maturation. This is believed to reflect the maturation of internal predictive (forward) models that link motor actions to their sensory consequences (Tanaka et al. 2019). However, the capacity to leverage such predictions for goal‐directed behavior—such as selecting actions based on expected outcomes—emerges more gradually over time. Developmental research also highlights improvements in performance monitoring throughout adolescence (Hogan et al. 2005; Ladouceur et al. 2007). These include heightened sensitivity to feedback, better error detection, and greater behavioral flexibility (Tamnes et al. 2013). Neurophysiologically, this behavioral trajectory corresponds with protracted maturation of the anterior cingulate cortex (ACC), a region implicated in action monitoring and error detection. The amplitude of the error‐related negativity (ERN), an event‐related potential generated in the ACC, increases from childhood through young adulthood, indexing enhanced internal monitoring processes (Hogan et al. 2005; Tamnes et al. 2013). Concurrently, structural and functional development in frontal and parietal cortices supports a shift from reactive, stimulus‐driven responses to more proactive, model‐based control strategies—where behavior is guided by internal models of action‐outcome contingencies rather than immediate environmental cues (Scholz et al. 2023).

However, brain maturation is not solely reflected in changes to the activity of existing adult‐like networks; rather, the underlying network structure itself may be qualitatively different, with additional brain regions being recruited. Considering the developing nature of adolescents' AEB‐related circuitry, the neural network supporting AEB processes in adults (Mayer, Mückschel, et al. 2025; Mayer, Helin Koyun, et al. 2025) may not only be altered in the way these structures communicate with one another—but may also see additional cortical structures come into play. For AEB, these additional cortical structures could refer to superior parietal cortices and ventral stream cortical structures. A few studies have indeed provided evidence for a role of the ventral stream during AEBs (Elsner and Hommel 2001; Kühn et al. 2011; Melcher et al. 2008, 2013). The ventral stream plays a crucial role in the integration of sensory information with motor actions (Eggert et al. 2023). During adolescence, the ventral visual stream undergoes structural and functional reorganization (Mills et al. 2014; Vos De Wael et al. 2021; Walhovd et al. 2015). Likewise, superior parietal structures undergo substantial developmental changes (Arnsten and Rubia 2012) crucial for perception‐action integration (Gottlieb and Snyder 2010), including processes outlined by ideomotor theory (Gholamipourbarogh et al. 2023). Taken together, adolescents' AEB‐related neural circuitry is still in development, potentially engaging a more distributed and functionally flexible network. Therefore, in addition to the cortical networks underlying AEB described previously (Mayer, Mückschel, et al. 2025), the aforementioned structures may also contribute to AEB processes in adolescence.

To investigate these questions, we employed an EEG frequency tagging technique to track the activation of action‐effect representations over time. This method leverages the brain's ability to synchronize to specific frequencies (e.g., flickering stimuli), allowing us to “tag” neural responses linked to learned action‐effects. Following the acquisition of an action‐effect, increased brain activity at the frequency corresponding to the previously entrained action‐effect was observed, indicating anticipatory activation of the action‐effects before carrying out an action. Given the established role of theta‐band oscillations in AEB, we focused on 4.5 Hz stimulation, —within the lower theta range (4–7 Hz)—which is closely associated with long‐range cortical communication and cognitive control (Mayer, Helin Koyun, et al. 2025; Mayer, Mückschel, et al. 2025). To ensure specificity, we included an 8 Hz control condition just above the theta range as a control condition. However, our primary focus was on investigating the neural interactions between the components of AEBs during action control, rather than the frequency tagging effect itself. To directly assess age‐related differences, we compared adolescent EEG data collected for this study with adult data from Mayer, Helin Koyun, et al. (2025), Mayer, Mückschel, et al. 2025, applying an identical preprocessing pipeline, beamforming strategy, and temporal segmentation protocol. This methodological consistency enabled a precise comparison of AEB‐related neural dynamics across age groups. Our primary focus was to examine the directed network communication underlying action–effect binding and retrieval, and how these processes differ between adolescents and adults. To evaluate the complex interplay of AEB processes in adolescents compared to adults, we used nonlinear Causal Relationship Estimation by Artificial Neural Network (nCREANN) to investigate the directed communication between cortical regions associated with theta band activity (Elmers et al. 2024; Ghorbani et al. 2024; Jamous et al. 2024; Mayer, Mückschel, et al. 2025; Wang et al. 2024).

While our approach is exploratory, we hypothesize that the canonical IC–ATL–IFC network will be present in both groups but show developmental differences in directed connectivity strength and anatomical distribution, with potential consolidation from cortical structures within the ventral stream in adolescents.

## 2 Materials and Methods

### 2.1 Participants

Two groups were tested in the experiment. The adult group consisted of *n* = 31 datasets (18 female; 25.14 ± 3.60 years; range 19–39 years) for final analysis. The adult sample is identical to the sample used in Mayer, Helin Koyun, et al. (2025) and Mayer, Mückschel, et al. (2025). In the adolescent group, *n* = 29 datasets (14 female; 13.3 ± 1.9 years; range 10–16 years) were included in the final analysis. Three datasets were excluded due to erroneous EEG recordings, one dataset due to insufficient data quality, and one dataset due to processing errors during nCREANN analysis. All participants were screened for psychiatric and neurological disorders and met the following criteria: no history of cardiovascular disease, self‐reported alcohol use below 16 drinks per week, no use of psychoactive medication, no mental or physical disabilities that could hinder participation, and no history of brain surgery. Informed written consent was obtained from all participants. In the case of the underaged group, written consent was also given by their legal guardian. The experiment received ethical approval from the Ethics Committee of the TU Dresden and followed the guidelines of the Declaration of Helsinki.

### 2.2 Task

**FIGURE 1:**
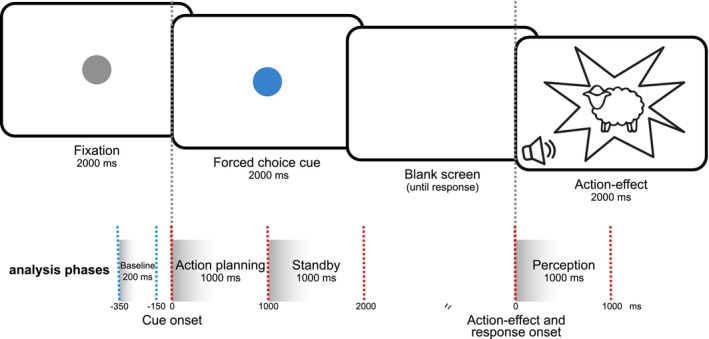
Experimental setup. Schematic depiction of the action‐effect binding experiment. The timing of all stimuli is depicted. Blue dashed lines indicate the baseline interval relative to the onset of the cue stimulus. Red dashed lines show the time interval of three analysis time windows, either relative to the onset of the cue (“action planning” phase, “standby” phase) or to the action‐effect (“perception” phase). The scaling of the time axis is not proportional.

The study employed a forced‐choice frequency‐tagging task, depicted in Figure 1 (Dignath et al. 2020; Elsner and Hommel 2001; Mayer, Helin Koyun, et al. 2025; Mayer, Mückschel, et al. 2025).

Action‐effect associations were learned by linking a specific keypress to a visual stimulus and a corresponding sound. The visual stimulus featured flickering images of either a cartoon‐style sheep or cat, with flicker frequencies of 4.5 or 8 Hz. The associated sound—a sheep or cat call—was played through headphones at 55 dB. Each trial began with a grey circle (1 cm radius) displayed at the center of the screen. After 2000 ms, the circle changed color to either blue or red, serving as the cue stimulus, and remained visible for another 2000 ms. Participants were instructed to press either the right or left CTRL key depending on the dot's color, doing so after the stimulus disappeared and within a 1000 ms response window. A correct response immediately triggered the visual and auditory action‐effect for 2000 ms, while incorrect, late, or missing responses resulted in no action‐effect presentation. The visual action‐effect was expected to elicit modulation of the steady‐state visual evoked potential (SSVEP) power in the EEG at the tagged frequency and to persist during the anticipation of the expected action‐effect following cue onset. This sustained activity would indicate successful acquisition and retrieval of action‐effect contingencies, in line with Mayer, Mückschel, et al. (2025). To ensure balanced distribution, stimulus‐key pairings were randomized across participants, creating 16 unique experimental conditions based on cue color (red/blue), response (left/right), action‐effect (cat/sheep), and flicker frequency (4.5/8 Hz). However, these conditions remained consistent for each participant across both sessions. The first session included 30 practice trials to establish action‐effect associations before the main experiment, which consisted of 300 randomized trials—150 for each flicker frequency. The experiment was conducted using the stimulus presentation software “Presentation” (version 18.3, Neurobehavioral Systems Inc., Berkeley, CA, www.neurobs.com). Participants were seated in front of a 24‐in. TFT.

### 2.3 EEG Recording and Pre‐Processing

EEG data were recorded using QuickAmp and BrainAmp amplifiers (Brain Products GmbH, Gilching, Germany) with 60 Ag‐AgCl electrodes in an equidistant setup with a 500 Hz sampling rate (reference electrode at Fpz). Impedances were kept below 5 kΩ. All processing steps were done using the preprocessing toolbox Automagic (Pedroni et al. 2019) and EEGLAB (Delorme and Makeig 2004) on Matlab (2021b, The MathWorks Corp). Preprocessing started with downsampling to 256 Hz. Channels used for EOG regression (AF7, AF8, Fp1, Fp2) were analyzed for flat segments (activity below 5 μV for 10 s) and, if affected, interpolated (spline interpolation). For artifact removal, the PREP preprocessing pipeline (Bigdely‐Shamlo et al. 2015) was used. The PREP pipeline applies a multitaper algorithm to eliminate 50 Hz line noise and subsequently implements a resilient average reference following the removal of artifacts from bad channels. Next, the EEGLAB clean_rawdata() pipeline was applied, including a FIR high‐pass filter of 0.5 Hz (order 1286, stop‐band attenuation 80 dB, transition band 0.25–0.75 Hz). Flat lines, noisy channels, and outlier channels were identified and eliminated. Epochs displaying unusually high power (> 15 standard deviations compared to calibration data) were reconstructed utilizing Artifact Subspace Reconstruction (ASR; burst criterion: 15; Mullen et al. 2013). Time windows that could not be reconstructed were excluded. A lowpass filter of 40 Hz was implemented using a sinc FIR filter with an order of 86 (Widmann et al. 2015). EOG artifacts were mitigated through an EOG regression method (Parra et al. 2005). Artifact removal of loose electrodes, muscle artifacts, and any remaining eye artifacts was done by an Independent Component Analysis (ICA) with an automatic labeling of the components using Multiple Artifact Rejection Algorithm (MARA; Winkler et al. 2014, 2011). For MARA, a temporary high‐pass FIR filter was applied (pop_eegfiltnew(), passband edge(s): 1 Hz, filter order: 846, transition band width: 1 Hz). Additionally, components containing cardiac artifacts were identified using ICLabel (threshold: 0.8; Pion‐Tonachini et al. 2019) and subsequently eliminated. On average, MARA and ICLabel rejected 19.5 components (±6.7; range 0–36). All removed channels underwent interpolation using a spherical method. On average, 10.3 channels (±4.1; range 4–20) were removed, including the 4 channels used for EOG regression.

### 2.4 Time‐Frequency Decomposition and EEG Beamformer

Brain vision analyzer (version 2.1, Brain Products GmBH, Gliching, Germany) was used to segment data in epochs of (i) −2000 to 4000 ms relative to the cue stimulus (cue‐locked) and (ii) −2000 to 4000 ms relative to the response or onset of the action‐effect (action‐effect locked), for both action‐effect flicker frequencies (4.5 and 8 Hz). As described in the Introduction, 8 Hz was included as a control frequency outside the theta range to contrast theta‐specific entrainment effects elicited by 4.5 Hz flicker stimulation. This contrast allows us to isolate the role of theta activity in AEB mechanisms.

Subsequent data analysis was performed with the FieldTrip Toolbox (Oostenveld et al. 2010) in Matlab. Time‐frequency (TF) decomposition was performed utilizing Morlet wavelets (number of wavelet cycles: 5; wavelets lengths: 3) for cue‐locked as well as action‐effect locked data. Relative power changes of response‐locked epochs were calculated on baseline normalized data (Dignath et al. 2020). The mean power of the 200 ms stimulus‐locked baseline interval (−350 to −150 ms) was computed and subtracted from each power value of the response‐locked epochs, and the differences were divided by the mean baseline power.

To isolate effects specific to theta‐band entrainment, power at the control frequency (8 Hz) was subtracted from power at the target frequency (4.5 Hz). This subtraction controlled for non‐specific effects of frequency tagging, thereby enhancing sensitivity to entrained neural dynamics specifically associated with AEB processes, such as anticipatory action planning and perceptual integration.

To ensure consistency in temporal segmentation across age groups, we verified whether the predefined cognitive intervals—derived from adult data—were applicable to adolescent participants. Visual inspection of relative power patterns confirmed clear and consistent phase‐specific activity in both groups. Although adolescents showed a modest delay in theta‐band activity onset, the overall temporal structure of cognitive phase progression was preserved, supporting the use of a uniform segmentation scheme across age groups.

In line with Mayer, Helin Koyun, et al. (2025) and Mayer, Mückschel, et al. (2025), further analysis was focused on 4.5 Hz induced flicker frequency. Three successive time windows (phases) were defined: the “action planning” phase from 0 to 1000 ms (cue‐locked), the “standby” phase from 1000 to 2000 ms (cue‐locked), and the “perception” phase from 0 to 1000 ms (action‐effect‐locked). It is assumed that each phase is associated with specific cognitive processes. Starting with the action planning phase from cue onset at 0 until 1000 ms, processes related to perceptual processing and response selection should be evident. These are typically characterized by the N1‐N2‐P3 event‐related potential complex (e.g., Folstein and Van Petten 2008; Luck 2005; Näätänen and Picton 1987; Polich 2007) and theta band activity (Cavanagh and Frank 2014; Nigbur et al. 2011) within the first 600 ms. To ensure robust frequency resolution, a length of 1000 ms was chosen for this phase. Next, it is assumed that in the “standby” phase (1000–2000 ms relative to cue onset) response selection processes have finished. Waiting for the go signal, response execution remains on hold. The perception phase (0–1000 ms action‐effect‐locked) includes the perceptual processing of the action effect, the binding of perceptual features, and post‐response evaluation (e.g., Danielmeier and Ullsperger 2011; Falkenstein et al. 2000; Miyake and Friedman 2012). To ensure comparability, the length of all three phases was kept similar.

### 2.5 DICS Beamformer

Source activity was reconstructed for each phase and group using dynamic imaging of coherent sources DICS beamforming (Gross et al. 2001). A forward model utilizing the Montreal Neurological Institute (MNI) brain template (Collins et al. 1998) facilitated the projection of localized brain activity from the DICS beamformer into a source space onto an evenly spaced grid (Oostenveld et al. 2010). Spectral analysis was conducted using a Hanning taper to compute power and the cross‐spectral density matrix at frequencies 4–5 Hz. Following the alignment of EEG electrodes with the forward model, a leadfield matrix was computed by dividing the brain volume into a grid with a 5 mm resolution. The leadfield matrix was then calculated for each grid point. A common spatial filter was applied across all phases, incorporating a regularization parameter set at 5%. The source estimation underwent normalization by an estimate of spatially inhomogeneous noise for each voxel to correct for noise bias towards the center of the head (Van Veen et al. 1997). The noise estimate was computed based on the smallest eigenvalue of the cross‐spectral density matrix.

### 2.6 Selection of Regions of Interest (ROI) and LCMV Beamformer

The selection of regions of interest (ROIs) for consecutive nCREANN analysis was predicated on outcomes from the DICS beamformer. Initially, all voxels within 2% proximity to the highest source activity values within functional neuroanatomical regions were chosen. Subsequently, these voxels underwent clustering using the Density‐Based Spatial Clustering of Applications with Noise (DBSCAN) algorithm (Adelhöfer et al. 2020; Ester et al. 1996). DBSCAN facilitates the identification of irregularly shaped clusters within distributed data points, eliminating the need for a predefined number of clusters. An epsilon value equivalent to twice the edge length of each voxel was selected to ensure the detection of neighboring voxels.

Based on the resultant clusters and associated Automated Anatomical Labeling (AAL) atlas labels (Rolls et al. 2020), ROIs were designated for each phase (i.e., Action Planning, Standby, Perception) for each group individually. Virtual neural time series were computed for every voxel within each ROI and period using the Linearly Constrained Minimum Variance (LCMV) beamformer (Van Veen et al. 1997). The time series for each voxel was derived by multiplying the LCMV spatial filter with the time‐domain time series. Subsequently, Time‐Frequency (TF) analysis was performed on the resulting time series using Morlet wavelets at 4.5 Hz. Power values were then averaged across voxels, delineated for each phase and ROI.

### 2.7 Non‐Linear Causal Relationship Estimation by Artificial Neural Networks (nCREANN)

**FIGURE 2:**
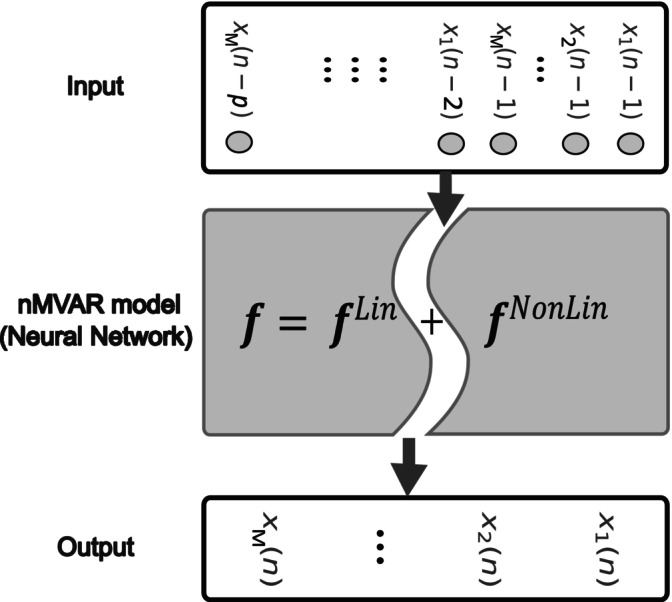
nCREANN flowchart. A feed‐forward neural network designed to model interactions among the *M* regions. The network receives lagged samples of the *M* signals as input ($x_{p}$). During training, it predicts future samples at the outputs ($x\left(n\right)$), based on past observations from all regions. The underlying function f, modeled by the network, is divided into two components: A linear part ($f^{\mathrm{Lin}}$) and a nonlinear part ($f^{\text{NonLin}}$). This separation enables the identification of both linear and nonlinear components of directed connectivity between the regions.

nCREANN (nonlinear Causal Relationship Estimation by Artificial Neural Network) was employed to assess connectivity patterns within the brain utilizing artificial neural networks (ANNs) for the estimation of effective connectivity among multiple brain regions. This approach builds upon a nonlinear Multivariate Autoregressive (MVAR) model, where the interactions between brain regions at current time points are influenced by their prior activities. Figure 2 provides a schematic representation of the nCREANN model, illustrating how lagged signals are used to predict future samples, with both linear and nonlinear components explicitly modeled.

The nCREANN model is represented by the equation:

(1) $$x\left(n\right)=f\left(x_{p}\right)+\sigma \left(n\right)$$

where $x_{p}={\left[x_{1}\left(n-1\right),x_{2}\left(n-1\right),\cdots ,x_{M}\left(n-p\right)\;\right]}^{T}$ denotes the vector of *p* previous samples of *M* time series and $\sigma \left(n\right)={\left[\sigma_{1},\sigma_{2},\ldots ,\sigma_{M}\;\right]}^{T}$ represents the residual of the model.

nCREANN captures linear and nonlinear dynamics of information flow among brain regions. This is particularly important, as brain interactions often exhibit high nonlinearity, and linear methods may oversimplify the intricate functions of the brain. Previous studies have highlighted the necessity of incorporating both linear and nonlinear concepts for a more comprehensive understanding of neurodynamics at a macroscale (Chen et al. 2009; Cifre et al. 2021; Ferdousi et al. 2020; Friston 2001; Nozari et al. 2020).

In nCREANN, the nMVAR model is implemented using a single‐hidden‐layer feed‐forward neural network. This network employs nonlinear activation functions for hidden neurons and linear functions at the output layer. To extract both linear and nonlinear information embedded within the network's structure, the linear $\left(f^{\mathrm{Lin}}\right)$ and nonlinear $f^{\text{NonLin}}$ parts of the network input–output mapping $f\left(.\right)$ Are separated:

(2) $$f=f^{\mathrm{Lin}}+f^{\text{NonLin}}$$

This separation is achieved by dividing the linear and nonlinear parts of the hidden neurons' functions assessed by their corresponding Taylor expansion.

To compute linear effective connectivity $\left({\mathrm{lC}}_{i\to j}\right)$, the approach involves $f^{\mathrm{Lin}}$ and is determining it by multiplying the network's connection weights with the scaling parameters of the hidden neurons. This measure signifies the degree to which the *i*th input node linearly influences the *j*th output neuron.

Nonlinear effective connectivity (${\mathrm{NC}}_{i\to j}$) from $x_{i}$ to $x_{j}$ is defined as the ratio of the network's estimation errors:

(3) $${\mathrm{NC}}_{i\to j}=\ln\left(\frac{\left\langle {{\left(\epsilon_{j}\right)}_{x_{i}\_\mathrm{Lin}}}^{2}\right\rangle }{\left\langle {\left(\epsilon_{j}\right)}^{2}\right\rangle }\right)$$

Here, the numerator's estimation error pertains to scenarios where $x_{i}$ contributes to $x_{j}$ is defined purely linearly, while the denominator's error corresponds to situations where all input signals exert both linear and nonlinear effects on $x_{j}$. ${\mathrm{NC}}_{i\to j}$ quantifies the extent of the nonlinear causal effect of $x_{i}$ on $x_{j}$. The optimal model order was calculated for each group individually and Schwarz Bayesian Criterion (adults = 20, adolecents = 8) was applied in the ARfit toolbox (Multivariate Autoregressive Model Fitting; Neumaier and Schneider 2001; Schneider and Neumaier 2001). A Multilayer Perceptron neural network with 1 hidden layer and 6 hidden neurons is trained using the gradient descent error back‐propagation (EBP) algorithm with momentum (*α*) and adaptive learning rate (*η*). Early stopping is applied for generalization, and a 5‐fold permuted cross‐validation is employed, with data split into 80% training, 10% validation, and 10% testing sets. The ‘incremental mode’ with random initial parameters in the range of −0.5 to 0.5 was used to update the network. Connectivity patterns are visualized on a head model, where arrows indicate information flow between clusters of sources. Arrow thickness is proportional to the strength of connectivity. Model evaluation is conducted using mean square error (MSE) and the coefficient of determination (*R* ^2^) for both training and test data. *R* ^2^ values, ranging from 0 (worst fit) to 1 (best fit), highlight the goodness of regression models, emphasizing appropriate generalization when similar values are observed for training and test sets. Additionally, the significance of connectivity values is assessed using a randomization test with 100 data sets generated through a time‐shifted surrogate technique (Papana et al. 2013). It is noteworthy that the network settings remain identical for both the original and surrogate data, ensuring robust comparison.

### 2.8 Quantification and Statistical Analysis

To test for differences between behavioral measures (mean RTs and accuracy) between the adult group and the adolescent group, an independent sample t‐test was applied. Additionally, Bayes factor BF_10_ was computed. BF_10_ values were interpreted as follows: Evidence towards H1: anecdotal 1–3; moderate 3–10; strong 10–30; very strong 30–100; extreme > 100. Evidence towards H0: anecdotal 0.33–1; moderate 0.1–0.33; strong 0.03–0.1; very strong 0.01–0.03; extreme < 0.01 (Lee and Wagenmakers 2014). Alpha was set to 0.05 if not specified otherwise. To show the modulatory effect of the action‐effect flicker at the respective frequencies, average power values in the perception phase were compared between action‐effect frequencies by means of repeated measures ANOVAs. Relative power changes for each datapoint were compared by means of paired‐sample *t*‐tests. FDR correction (Genovese et al. 2002) was applied to account for multiple testing (*α* = 0.05).

To evaluate asymmetries in connectivity values between regions of interest (ROIs), a paired‐sample t‐test was performed on the bidirectional connection pairs. FDR correction was applied to account for multiple testing (*α* = 0.05). Additionally, Bayes factor BF_10_ was computed. To compare linear and non‐linear connectivities between the two groups, independent‐sample *t*‐tests were conducted, and FDR correction was applied. Only regions defined for both groups were compared (i.e., ATL, IC, IFC). Additionally, Bayes factor BF_10_ was computed.

## 3 Results

### 3.1 Behavioral Data

Regarding the reaction times (RTs), participants in the adult group on average showed faster RTs (338.27 ± 14.04) in comparison to the adolescent group (376.69 ± 11.62), as indicated by a significant independent samples *t*‐test (*t*(58) = −2.09; *p* = 0.041; *d* = −0.54). A Bayesian test revealed anecdotal evidence towards H1 (BF_10_ = 2.52). Regarding response accuracy, there was no significant difference for the number of correct responses (*t*(58) = 0.84; *p* = 0.403; *d* = 0.22) between the adult group (0.95 ± 0.07) and the adolescent group (0.93 ± 0.06). A Bayesian test showed anecdotal evidence towards H0 (BF_10_ = 0.5).

### 3.2 Neurophysiological Data

**FIGURE 3:**
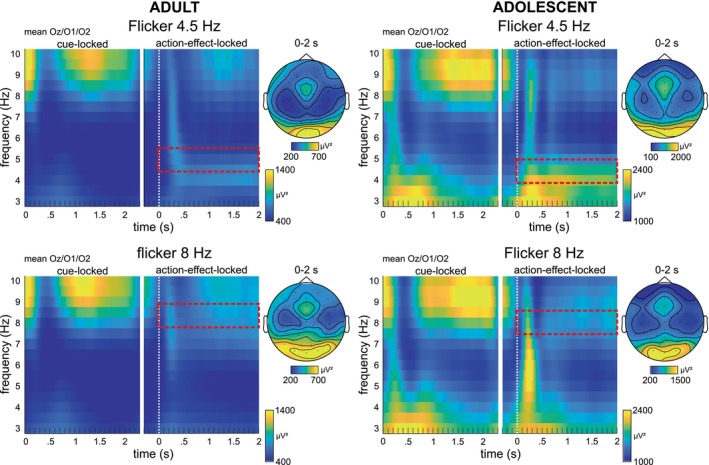
Time‐frequency results. Time‐frequency power of the action‐effect flicker frequency at 4.5 and 8 Hz for adult group (left) and adolescent group (right). Averaged power values of electrodes Oz, O1, and O2 are presented. The time window is 0–2.3 s relative to the onset of the cue‐stimulus as well as −0.3 to 2 s relative to the action‐effect onset (indicated by white dotted line). The topography plots show the power distribution at the respective action‐effect frequency averaged from 0 to 2 s relative to action‐effect onset (red dashed box).

Figure 3 illustrates the average power modulations observed at averaged electrodes Oz, O1, and O2 throughout the experiment for the action‐effect flicker frequencies of 4.5 and 8 Hz, for the adult group (left) and for the adolescent group (right). To analyze the modulations by the action‐effect flicker, two repeated measure ANOVAs were computed for the mean power of electrodes Oz, O1, and O2 in the time window of the action‐effect of 0 to 2000 ms relative to the action‐effect onset. The first ANOVA analyzed the effect of the two different flicker frequencies on the power at 4.5 Hz. The ANOVA showed a main effect of action‐effect flicker frequency (*F*(1, 58) = 48.54; *p* < 0.001; *η* ^2^ *p* = 0.46), indicating that overall the power at 4.5 Hz was higher for an action‐effect of 4.5 Hz compared to an action‐effect of 8 Hz. Additionally, there was a significant main effect of group (*F*(1, 58) = 98.52; *p* < 0.001; *η* ^2^ *p* = 0.63), indicating that the power at 4.5 Hz was higher in the adolescent group in comparison to the adult group. As shown by post hoc pairwise comparisons, the adolescent group showed higher power modulations at 4.5 Hz for an action‐effect of 4.5 Hz (1808.2 ± 177.9) compared to the modulations by an action‐effect of 8 Hz (1496.2 ± 144.7; *p* < 0.001). A similar pattern emerged for the adult group; the power at 4.5 Hz was significantly higher for an action‐effect of 4.5 Hz (677.7 ± 172) in comparison to 8 Hz (421.3 ± 139.9). The second ANOVA investigated the modulations at 8 Hz power. There was a significant main effect of action‐effect flicker frequency (*F*(1, 58) = 22.6; *p* < 0.001; *η* ^2^ *p* = 0.28), indicating that for both groups power at 8 Hz was higher for an action‐effect of 8 Hz in comparison to 4.5 Hz. Additionally, the significant main effect of group (*F*(1, 58) = 80.39; *p* = 0.001; *η* ^2^ *p* = 0.17) indicates that the overall power at 8 Hz was higher for adolescents compared to adults. For adolescents, pairwise comparisons showed significantly higher power at 8 Hz for an action‐effect of 8 Hz (1511.9 ± 171.3) compared to 4.5 Hz (1369.8 ± 165.7). For adults, the power at 8 Hz again was larger for an action‐effect of 8 Hz (716 ± 165.7) compared to 4.5 Hz (572.6 ± 160.3).

**FIGURE 4:**
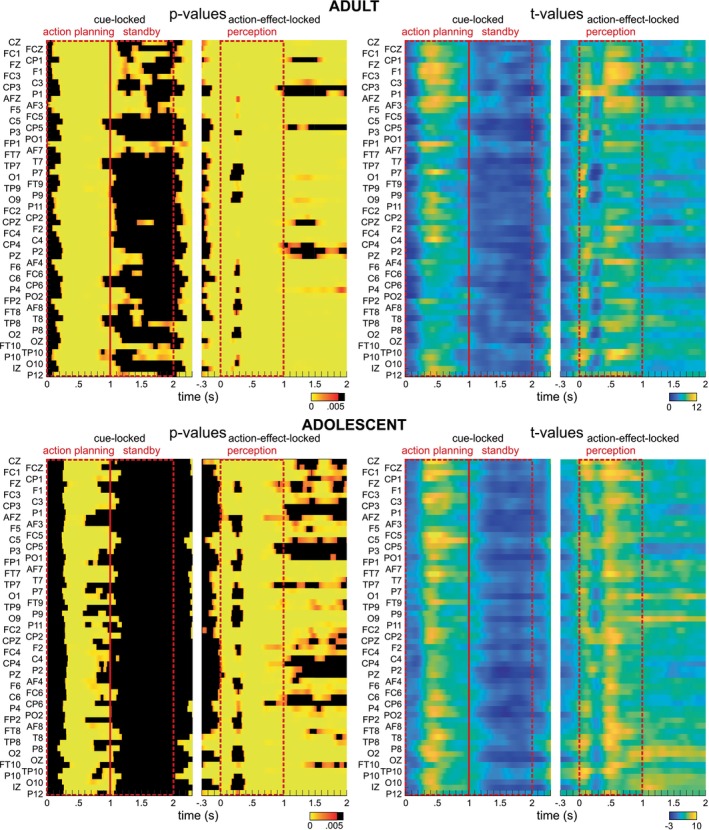
Relative power changes. Results of t‐tests comparing relative power changes at 4.5 and 8 Hz (i.e., measured frequencies) at a flicker frequency 4.5 Hz, for the adult group (top) and the adolescent group (bottom). For cue‐locked data, all time‐points between 0 and 2.3 s were compared, for action‐effect‐locked data the time window was −0.3 to 2 s. *p* values (left) and *t* values (right) are depicted by colors, non‐significant *p* values after FDR correction are indicated by black areas. The data was segmented into three different analysis phases: The “action planning” and the “standby” phase relative to the cue stimulus onset, and the “perception phase” relative to the action‐effect onset.

Following the analytical approach of Mayer, Helin Koyun, et al. (2025) and Mayer, Mückschel, et al. (2025), the focus was placed on the 4.5 Hz flicker frequency data. Relative power changes were compared between 4.5 and 8 Hz. Paired‐sample t‐tests were conducted for each data point and electrode. For cue‐locked data, this analysis covered all time points from 0 to 2300 ms after cue stimulus onset, while for the action‐effect‐locked data, a time window from −300 to 2000 ms relative to the action‐effect onset was used. FDR correction (Benjamini and Hochberg 1995) was applied to account for multiple testing (*α* = 0.05). Figure 4 depicts the results for the adult group on the top row; the results for the adolescent group are depicted below. For the adult group, the visual inspection of significant time points (all significant *p* values ≤ 0.003; t ≥ 3.24) revealed notable power changes at all electrodes, beginning around approximately 150 ms after cue stimulus onset (cue‐locked segmentation). These effects persisted across most electrodes until approximately 1000 ms. The time window from 1000 ms until the offset of the cue stimulus at 2000 ms showed remarkably less relative power change differences. In the action‐effect‐locked segmentation, significant changes were observed at all electrodes with the onset of the action‐effect/response at 0 ms until the end of the 2000 ms action‐effect period. For the adolescent group, significant differences of relative power changes (all significant *p* values ≤ 0.004; t ≥ 3.19) emerged starting at about 250 ms relative to the cue onset and diminishing from about 700 ms until 1000 ms. Almost no significant time points were evident between 1000 and 2000 ms. In the action‐effect‐locked segmentation, significant differences were observed between 0 and 2000 ms, though less pronounced in the time window from 1000 to 2000 ms. These findings provide important insights into the nature of the observed theta activity, particularly during the action‐effect/perception phase. While the SSVEP reflects stimulus‐evoked responses to flicker, the sustained theta activity during action planning in both groups points to an additional role for internally generated, anticipatory mechanisms associated with ideomotor processing—consistent with prior theoretical accounts.

In line with Mayer, Helin Koyun, et al. (2025) and Mayer, Mückschel, et al. (2025) and as described in the Methods section, the time course was divided into three consecutive 1000 ms intervals corresponding to distinct cognitive phases: the action planning phase (0–1000 ms, cue‐locked), the standby phase (1000–2000 ms, cue‐locked), and the perception phase (0–1000 ms, action‐effect‐locked; see Figures 1 and 4).

**FIGURE 5:**
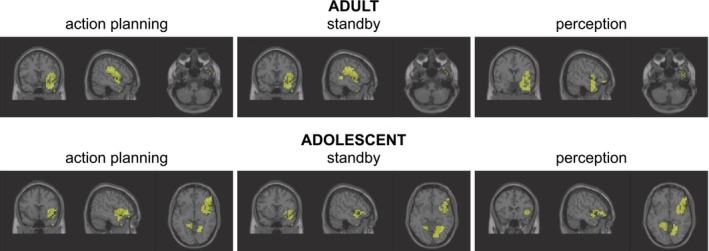
DICS source localization. DICS beamformer results at 4.5 Hz and action‐effect of 4.5 Hz for the adult group (top) and the adolescent group (bottom). Only the top 3% of voxels by source activity are shown. In the adult group, the beamformer revealed predominantly right‐hemisphere sources in temporal, inferior frontal areas and insular areas. In the adolescent group, also sources in the lingual gyrus were revealed.

A DICS beamformer was used to identify the cortical sources generating theta band activity at 4.5 Hz, separately for each analysis phase and group, as described in the Methods section. For each phase, the top 3% of voxels contributing most to theta band activity were selected, and their corresponding anatomical labels were determined. This voxel thresholding was applied consistently to both the adult and adolescent samples. By using the same data‐driven approach across groups, we ensured analytical consistency and enabled the identification of underlying network dynamics without imposing predefined anatomical constraints or a group‐dependent bias in thresholding that may affect the interpretation of the data. Voxels located outside the brain or within white matter were excluded, along with those within cerebellar structures. A DBSCAN clustering algorithm was then applied to group the selected voxels into clusters, which are visualized in Figure 5 for both groups. Next, the selected voxels were segmented manually. Individual voxels that were not assigned to any clusters were excluded. Furthermore, all voxels within subcortical nuclear structures, such as the basal ganglia, were excluded due to the inherent limitations of EEG source estimation, particularly for smaller and deeper brain structures. For the adult group, three distinct functional ROIs in the right hemisphere were determined: anterior temporal (ATL), inferior frontal (IFC) and insular (IC) areas. The ATL encompassed right‐hemisphere voxels with labels corresponding to inferior temporal gyrus, temporal pole (middle temporal gyrus), superior temporal gyrus, middle temporal gyrus, and temporal pole (superior temporal gyrus), based on the Automated Anatomical Labeling (AAL) atlas version 3 (Rolls et al. 2020). For the IC, voxels corresponding to the insula were included. For the IFC, the following areas were included: opercular part of inferior frontal gyrus, pars orbitalis of inferior frontal gyrus, and, except for the perception phase, rolandic operculum.

For the adolescent group, four distinct functional ROIs in the right hemisphere were defined: ATL, IC, IFC, and Lingual Gyrus (LG). Alike the definition for the adult group, the ATL defined for the adolescent group comprised voxels within the inferior temporal gyrus, temporal pole (middle temporal gyrus), superior temporal gyrus, middle temporal gyrus, and temporal pole (superior temporal gyrus). For the IC, voxels corresponding to the insula were included. For the IFC, voxels in the following areas were included: opercular part of the inferior frontal gyrus, pars orbitalis of the inferior frontal gyrus, and rolandic operculum. For LG, voxels in the lingual gyrus were included.

### 3.3 Linear and Non‐Linear Effective Connectivity

To determine the linear and non‐linear effective connectivity between the reconstructed sources, nCREANN analysis (Elmers et al. 2024; Ghorbani et al. 2024; Mayer, Helin Koyun, et al. 2025; Mayer, Mückschel, et al. 2025; Talebi et al. 2019) was conducted. The models were properly trained, as indicated by the model evaluation scores. Training and testing errors were small, as shown by a mean square error (MSE) of 0.04 (±0.02) for training data and of 0.04 ± 0.03 for testing data. Additionally, the *R* ^2^ values indicate a high fit of the model, with a mean *R* ^2^ of 0.97 ± 0.06 for training and an R^2^ of 0.97 ± 0.06 for testing. The *R* ^2^ values of the training and testing phase were highly positively correlated (*r* > 0.85; *p* < 0.001).

**FIGURE 6:**
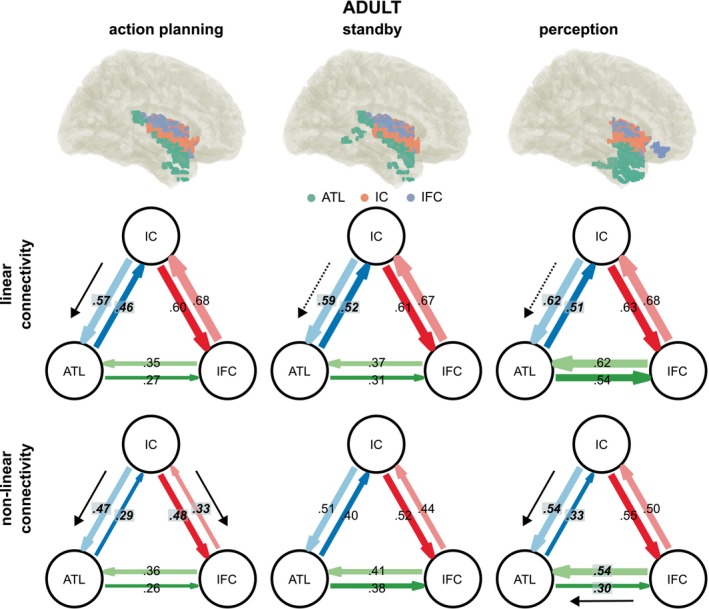
Connectivities adult group. Linear and non‐linear effective connectivity pattern of the adult group as revealed by nCREANN. For each phase, linear and non‐linear connectivity pattern between established ROIs (ATL, anterior temporal lobe; IC, insular cortex; IFC, inferior frontal cortex) are depicted. The direction of input flows between two ROIs is shown by colored arrows. The connectivity strengths are depicted by the thickness of the arrows. Significant differences between bidirectional connectivities after FDR correction (α = 0.05) are indicated by bold italic text. Additionally, black arrows indicate significant asymmetric connectivities. Dotted arrows indicate difference that did not survive FDR correction.

**FIGURE 7:**
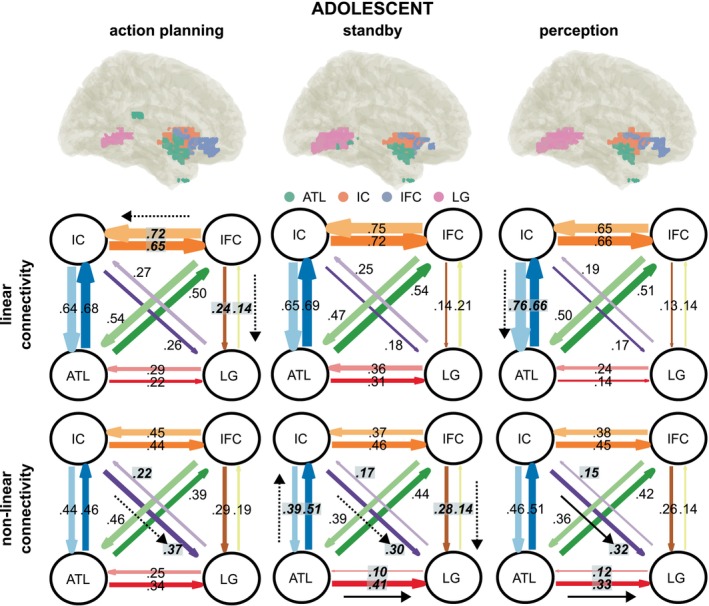
Connectivities adolescent group. Linear and non‐linear effective connectivity pattern of the adolescent group as revealed by nCREANN. For each phase, linear and non‐linear connectivity pattern between established ROIs (ATL, anterior temporal lobe; IC, insular cortex; IFC, inferior frontal cortex, LG, lingual gyrus) are depicted. The direction of input flows between two ROIs is shown by colored arrows. The connectivity strengths are depicted by the thickness of the arrows. Significant differences between bidirectional connectivities after FDR correction (*α* = 0.05) are indicated by bold italic text. Additionally, black arrows indicate significant asymmetric connectivities. Dotted arrows indicate difference that did not survive FDR correction.

Tables 1 and 2 provide detailed results of the nCREANN analysis, including the results of a paired‐sample frequentist and Bayesian t‐test comparing the bilateral connectivities for both groups. Linear and non‐linear connectivities for all three phases for the adult group are depicted in Figures 6 and 7 depicts the results for the adolescent group. Connectivity strength is depicted by the thickness of the arrows between anatomical regions. Bold text indicates significant differences in bilateral connectivities (*p* < 0.05) and black arrows indicate the predominant, asymmetric direction of information flow. FDR correction has been applied to account for multiple testing (*α* = 0.05) and differences that were not significant after FDR correction are indicated by dotted arrows.

**TABLE 1:** nCREANN results for the adult group.

Phase	Connection	ROI	ROI	*t*	*d*	*p*	*p* (FDR)	BF10	BF evidence
		a → b	b → a						
Linear									
Planning	ATL<>IC	0.46 ± 0.04	0.57 ± 0.05	−3.84	0.69	0.001	0.005	51.96	Very strong H1
Planning	ATL<>IFC	0.27 ± 0.03	0.35 ± 0.05	−1.81	0.32	0.080	0.137	0.81	Anecdotal H0
Planning	IC<>IFC	0.60 ± 0.05	0.68 ± 0.06	−1.97	0.35	0.058	0.130	1.05	Anecdotal H1
Standby	ATL<>IC	0.52 ± 0.05	0.59 ± 0.04	−2.13	0.38	0.042	0.125	1.36	Anecdotal H1
Standby	ATL<>IFC	0.31 ± 0.04	0.37 ± 0.04	−1.66	0.30	0.107	0.137	0.65	Anecdotal H0
Standby	IC<>IFC	0.61 ± 0.05	0.67 ± 0.05	−1.59	0.29	0.122	0.137	0.60	Anecdotal H0
Perception	ATL<>IC	0.51 ± 0.04	0.62 ± 0.04	−2.25	0.40	0.032	0.125	1.70	Anecdotal H1
Perception	ATL<>IFC	0.54 ± 0.04	0.62 ± 0.04	−1.59	0.29	0.122	0.137	0.59	Anecdotal H0
Perception	IC<>IFC	0.63 ± 0.04	0.68 ± 0.04	−1.39	0.25	0.176	0.176	0.46	Anecdotal H0
Non‐linear									
Planning	ATL<>IC	0.29 ± 0.05	0.47 ± 0.05	−2.82	0.51	0.008	0.025	5.19	Moderate H1
Planning	ATL<>IFC	0.26 ± 0.05	0.36 ± 0.05	−1.99	0.36	0.056	0.100	1.08	Anecdotal H1
Planning	IC<>IFC	0.48 ± 0.06	0.33 ± 0.05	2.58	0.46	0.015	0.034	3.15	Moderate H1
Standby	ATL<>IC	0.40 ± 0.05	0.51 ± 0.05	−1.76	0.32	0.088	0.132	0.76	Anecdotal H0
Standby	ATL<>IFC	0.38 ± 0.06	0.41 ± 0.05	−0.39	0.07	0.696	0.696	0.21	Moderate H0
Standby	IC<>IFC	0.52 ± 0.06	0.44 ± 0.05	1.39	0.25	0.175	0.224	0.46	Anecdotal H0
Perception	ATL<>IC	0.33 ± 0.05	0.54 ± 0.05	−3.38	0.61	0.002	0.009	17.55	Strong H1
Perception	ATL<>IFC	0.30 ± 0.04	0.54 ± 0.05	−5.12	0.92	0.000	0.000	1329.16	Extreme H1
Perception	IC<>IFC	0.55 ± 0.06	0.50 ± 0.05	0.84	0.15	0.406	0.457	0.27	Moderate H0

*Note:* Linear and non‐linear connectivity and results of frequentist and Bayesian *t*‐test. Mean and SEM are given for each connection beside *t* value (*t*), Cohen's *d* (*d*), *p* value (*p*) and FDR corrected *p* value (*p* (FDR)). The Bayes factor strength of evidence label is given as evidence towards H1 if BF10 > 1 and otherwise as evidence towards H0 based on Lee and Wagenmakers (2014).

**TABLE 2:** nCREANN results for the adolescent group.

Phase	Connection	ROI	ROI	*t*	*d*	*p*	*p* (FDR)	BF10	BF evidence
		a → b	b → a						
Linear									
Planning	ATL<>IC	0.68 ± 0.04	0.64 ± 0.04	0.98	0.18	0.334	0.467	0.31	Moderate H0
Planning	ATL<>IFC	0.50 ± 0.03	0.54 ± 0.04	−1.26	0.23	0.219	0.417	0.40	Anecdotal H0
Planning	ATL<>LG	0.22 ± 0.04	0.29 ± 0.05	−1.35	0.25	0.187	0.417	0.45	Anecdotal H0
Planning	IC<>IFC	0.65 ± 0.04	0.72 ± 0.04	−2.52	0.47	0.018	0.159	2.83	Anecdotal H1
Planning	IC<>LG	0.26 ± 0.04	0.27 ± 0.05	−0.11	0.02	0.914	0.914	0.20	Moderate H0
Planning	IFC<>LG	0.24 ± 0.04	0.14 ± 0.03	2.27	0.42	0.031	0.188	1.77	Anecdotal H1
Standby	ATL<>IC	0.69 ± 0.04	0.65 ± 0.03	0.98	0.18	0.337	0.467	0.30	Moderate H0
Standby	ATL<>IFC	0.54 ± 0.04	0.47 ± 0.05	1.32	0.24	0.199	0.417	0.43	Anecdotal H0
Standby	ATL<>LG	0.31 ± 0.04	0.36 ± 0.05	−1.16	0.22	0.255	0.417	0.36	Anecdotal H0
Standby	IC<>IFC	0.72 ± 0.03	0.75 ± 0.04	−1.23	0.23	0.230	0.417	0.39	Anecdotal H0
Standby	IC<>LG	0.18 ± 0.03	0.25 ± 0.05	−1.18	0.22	0.248	0.417	0.37	Anecdotal H0
Standby	IFC<>LG	0.14 ± 0.03	0.21 ± 0.04	−1.35	0.25	0.188	0.417	0.45	Anecdotal H0
Perception	ATL<>IC	0.66 ± 0.04	0.76 ± 0.03	−2.83	0.53	0.009	0.154	5.20	Moderate H1
Perception	ATL<>IFC	0.51 ± 0.05	0.50 ± 0.04	0.20	0.04	0.840	0.914	0.20	Moderate H0
Perception	ATL<>LG	0.14 ± 0.04	0.24 ± 0.05	−1.88	0.35	0.070	0.317	0.93	Anecdotal H0
Perception	IC<>IFC	0.66 ± 0.04	0.65 ± 0.05	0.35	0.06	0.731	0.914	0.21	Moderate H0
Perception	IC<>LG	0.17 ± 0.04	0.19 ± 0.04	−0.29	0.05	0.777	0.914	0.20	Moderate H0
Perception	IFC<>LG	0.13 ± 0.04	0.14 ± 0.03	−0.13	0.02	0.901	0.914	0.20	Moderate H0
Non‐linear									
Planning	ATL<>IC	0.46 ± 0.05	0.44 ± 0.05	0.30	0.06	0.764	0.802	0.21	Moderate H0
Planning	ATL<>IFC	0.39 ± 0.04	0.46 ± 0.05	−0.93	0.17	0.360	0.462	0.29	Moderate H0
Planning	ATL<>LG	0.34 ± 0.05	0.25 ± 0.05	1.38	0.26	0.180	0.294	0.46	Anecdotal H0
Planning	IC<>IFC	0.44 ± 0.04	0.45 ± 0.06	−0.25	0.05	0.802	0.802	0.20	Moderate H0
Planning	IC<>LG	0.37 ± 0.05	0.22 ± 0.06	2.56	0.48	0.016	0.073	3.05	Moderate H1
Planning	IFC<>LG	0.29 ± 0.04	0.19 ± 0.06	1.64	0.31	0.111	0.201	0.65	Anecdotal H0
Standby	ATL<>IC	0.51 ± 0.06	0.39 ± 0.06	2.27	0.42	0.031	0.080	1.77	Anecdotal H1
Standby	ATL<>IFC	0.44 ± 0.05	0.39 ± 0.05	0.78	0.15	0.440	0.495	0.26	Moderate H0
Standby	ATL<>LG	0.41 ± 0.05	0.10 ± 0.05	6.18	1.15	0.000	0.000	15751.90	Extreme H1
Standby	IC<>IFC	0.46 ± 0.07	0.37 ± 0.06	1.80	0.33	0.083	0.166	0.81	Anecdotal H0
Standby	IC<>LG	0.30 ± 0.04	0.17 ± 0.05	2.30	0.43	0.029	0.080	1.87	Anecdotal H1
Standby	IFC<>LG	0.28 ± 0.04	0.14 ± 0.05	2.27	0.42	0.031	0.080	1.79	Anecdotal H1
Perception	ATL<>IC	0.51 ± 0.05	0.46 ± 0.06	0.84	0.16	0.406	0.488	0.27	Moderate H0
Perception	ATL<>IFC	0.42 ± 0.05	0.36 ± 0.05	1.16	0.21	0.258	0.357	0.36	Anecdotal H0
Perception	ATL<>LG	0.33 ± 0.05	0.12 ± 0.04	3.76	0.70	0.001	0.007	41.20	Very strong H1
Perception	IC<>IFC	0.45 ± 0.06	0.38 ± 0.05	1.20	0.22	0.238	0.357	0.38	Anecdotal H0
Perception	IC<>LG	0.32 ± 0.05	0.15 ± 0.04	2.95	0.55	0.006	0.038	6.68	Moderate H1
Perception	IFC<>LG	0.26 ± 0.05	0.14 ± 0.04	2.05	0.38	0.050	0.113	1.21	Anecdotal H1

*Note:* Linear and non‐linear connectivity and results of frequentist and Bayesian *t*‐test. Mean and SEM are given for each connection beside *t* value (*t*), Cohen's *d* (*d*), *p* value (*p*) and FDR corrected *p* value (*p* (FDR)). The Bayes factor strength of evidence label is given as evidence towards H1 if BF10 > 1 and otherwise as evidence towards H0 based on Lee and Wagenmakers (2014).

For the adult group (see Table 1 and Figure 6), linear connectivities ranged between 0.27 and 0.68, with a mean of 0.53 (±0.13). In the action planning phase, IC to ATL (0.57) was significantly higher than ATL to IC (0.46). In contrast, no differences were observed between IFC to IC (0.68) and IC to IFC (0.6), as well as between ATL to IFC (0.27) and IFC to ATL (0.35). In the standby phase, IC to ATL (0.59) was significantly higher than ATL to IC (0.52), but not after FDR correction. No significant differences were observed between ATL to IFC (0.31) and IFC to ATL (0.37), as well as between IC to IFC (0.61) and IFC to IC (0.67). In the perception phase, ATL to IC (0.51) was significantly smaller than IC to ATL (0.62), but not after FDR correction. No significant differences were observed between IC to IFC (0.63) and IFC to IC (0.68), as well as between ATL to IFC (0.54) and IFC to ATL (0.62).

Non‐linear connectivities ranged from 0.26 to 0.55 with a mean value of 0.42 (±0.09). In the action planning phase, IC to ATL (0.47) was significantly larger than ATL to IC (0.29). IC to IFC (0.48) was significantly larger than IFC to IC (0.33). ATL to IFC (0.26) was significantly smaller than IFC to ATL (0.36), but this difference was not significant after FDR correction. The standby phase showed no significant differences, neither for ATL to IC (0.4) and IC to ATL (0.51), IC to IFC (0.52) and IFC to IC (0.44), nor ATL to IC (0.38) and IC to ATL (0.41). In the perception phase, IC to ATL (0.54) was significantly larger than ATL to IC (0.33). Additionally, IFC to ATL (0.54) was significantly larger than ATL to IFC (0.3). IC to IFC (0.55) and IFC to IC (0.5) did not differ significantly.

For the adolescent group (see Table 2 and Figure 7), linear connectivities ranged from 0.13 to 0.76, with a mean of 0.42 (±0.22). In the action planning phase, no significant differences were observed between ATL to IC (0.68) and IC to ATL (0.64), between ATL to IFC (0.50) and IFC to ATL (0.54), between ATL to LG (0.22) and LG to ATL (0.29), as well as between IC to LG (0.26) and LG to IC (0.27). IC to IFC (0.65) was significantly lower than IFC to IC (0.72), but not significant after FDR correction. IFC to LG (0.24) was significantly higher than LG to IFC (0.14), but not significant after FDR correction. In the standby phase, significant differences between bidirectional connectivities were not observed, neither between ATL to IC (0.69) and IC to ATL (0.65), between ATL to IFC (0.54) and IFC to ATL (0.47), between ATL to LG (0.31) and LG to ATL (0.36), between IC to IFC (0.72) and IFC to IC (0.75), between IC to LG (0.18) and LG to IC (0.25), nor between IFC to LG (0.14) and LG to IFC (0.21). In the perception phase, ATL to IC (0.66) was significantly lower than IC to ATL (0.76), *p* = 0.009 (uncorrected), but not significant after FDR correction. No significant differences were observed between ATL to IFC (0.51) and IFC to ATL (0.50), between ATL to LG (0.14) and LG to ATL (0.24), between IC to IFC (0.66) and IFC to IC (0.65), between IC to LG (0.17) and LG to IC (0.19), nor between IFC to LG (0.13) and LG to IFC (0.14).

Non‐linear connectivities in the adolescent groups ranged between 0.1 and 0.51, with a mean of 0.34 (± 0.12). In the action planning phase, there were no significant differences between ATL to IC (0.46) and IC to ATL (0.44), between ATL to IFC (0.39) and IFC to ATL (0.46), between ATL to LG (0.34) and LG to ATL (0.25), between IC to IFC (0.44) and IFC to IC (0.45), and between IFC to LG (0.29) and LG to IFC (0.19). IC to LG (0.37) was significantly higher than LG to IC (0.22), but not significant after FDR correction. In the standby phase, ATL to IC (0.51) was significantly higher than IC to ATL (0.39), but not significant after FDR correction. ATL to LG (0.41) was significantly higher than LG to ATL (0.10). IC to LG (0.30) was significantly higher than LG to IC (0.17), but not significant after FDR correction. IFC to LG (0.28) was significantly higher than LG to IFC (0.14), but not significant after FDR correction. No significant differences were observed between IC to IFC (0.46) and IFC to IC (0.37), as well as between ATL to IFC (0.44) and IFC to ATL (0.39). In the perception phase, ATL to LG (0.33) was significantly higher than LG to ATL (0.12). IC to LG (0.32) was significantly higher than LG to IC (0.15). IFC to LG (0.26) was significantly higher than LG to IFC (0.14), but not significant after FDR correction. No significant differences were observed between ATL to IC (0.51) and IC to ATL (0.46), between ATL to IFC (0.42) and IFC to ATL (0.36), as well as between IC to IFC (0.45) and IFC to IC (0.38).

Additionally, linear and non‐linear connectivities were compared between the adult and adolescent groups. Only connections between regions defined for both groups were compared (i.e., ATL, IC, IFC). Detailed results are given in Table 3. For linear connections in the action planning phase, significant differences after FDR correction were observed between groups for IFC to ATL, ATL to IC, and ATL to IFC. For the standby phase, significant differences could be found for ATL to IC and ATL to IFC. Lastly, the perception phase showed significant differences between the adult and adolescent groups for IC to ATL and ATL to IC. The differences between adults and adolescents in IFC–ATL connectivity did not survive FDR correction. Similarly, when examining non‐linear connectivity, the initially significant connections in the action planning phase from the ATL to the IC and IFC in both groups remained anecdotal, as they did not survive FDR correction. During the perception phase, initial significant connections from the IFC to the ATL and from the ATL to the IC also failed to survive FDR correction, resulting in no significant non‐linear differences between adults and adolescents.

**TABLE 3:** Results of t‐tests comparing connectivities between the adult and adolescent groups for each phase.

Phase	Connection	Adult	Adolescent	*t*	*d*	*p*	*p* (FDR)	BF10	BF evidence
Linear									
Planning	IC → ATL	0.57 ± 0.05	0.64 ± 0.04	−1.16	−0.30	0.251	0.377	0.45	Anecdotal H0
Planning	IFC → ATL	0.35 ± 0.05	0.54 ± 0.04	−3.02	−0.78	0.004	0.017 *	1.35	Strong H1
Planning	ATL → IC	0.46 ± 0.04	0.68 ± 0.04	−3.73	−0.96	0.000	0.003 *	62.65	Very strong H1
Planning	IFC → IC	0.68 ± 0.06	0.72 ± 0.04	−0.54	−0.14	0.591	0.612	0.30	Moderate H0
Planning	ATL → IFC	0.27 ± 0.03	0.50 ± 0.03	−4.87	−1.26	0.000	0.000 *	185.13	Extreme H1
Planning	IC → IFC	0.60 ± 0.05	0.65 ± 0.04	−0.73	−0.19	0.467	0.600	0.33	Moderate H0
Standby	IC → ATL	0.59 ± 0.04	0.65 ± 0.03	−1.02	−0.26	0.313	0.433	0.41	Anecdotal H0
Standby	IFC → ATL	0.37 ± 0.04	0.47 ± 0.05	−1.48	−0.38	0.144	0.259	0.66	Anecdotal H0
Standby	ATL → IC	0.52 ± 0.05	0.69 ± 0.04	−2.80	−0.72	0.007	0.025 *	6.30	Moderate H1
Standby	IFC → IC	0.67 ± 0.05	0.75 ± 0.04	−1.29	−0.33	0.204	0.334	0.52	Anecdotal H0
Standby	ATL → IFC	0.31 ± 0.04	0.54 ± 0.04	−4.31	−1.11	0.000	0.001 *	325.45	Extreme H1
Standby	IC → IFC	0.61 ± 0.05	0.72 ± 0.03	−1.84	−0.47	0.072	0.144	1.02	Anecdotal H1
Perception	IC → ATL	0.62 ± 0.04	0.76 ± 0.03	−2.74	−0.71	0.008	0.025 *	5.52	Moderate H1
Perception	IFC → ATL	0.62 ± 0.04	0.50 ± 0.04	2.13	0.55	0.037	0.084	1.70	Anecdotal H1
Perception	ATL → IC	0.51 ± 0.04	0.66 ± 0.04	−2.65	−0.68	0.010	0.027*	4.59	Moderate H1
Perception	IFC → IC	0.68 ± 0.04	0.65 ± 0.05	0.55	0.14	0.582	0.612	0.30	Moderate H0
Perception	ATL → IFC	0.54 ± 0.04	0.51 ± 0.05	0.59	0.15	0.557	0.612	0.30	Moderate H0
Perception	IC → IFC	0.63 ± 0.04	0.66 ± 0.04	−0.51	−0.13	0.612	0.612	0.29	Moderate H0
Non‐linear									
Planning	IC → ATL	0.47 ± 0.05	0.44 ± 0.05	0.38	0.10	0.704	0.746	0.28	Moderate H0
Planning	IFC → ATL	0.36 ± 0.05	0.46 ± 0.05	−1.32	−0.34	0.190	0.342	0.55	Anecdotal H0
Planning	ATL → IC	0.29 ± 0.05	0.46 ± 0.05	−2.35	−0.61	0.022	0.132	2.54	Anecdotal H1
Planning	IFC → IC	0.33 ± 0.05	0.45 ± 0.06	−1.65	−0.43	0.104	0.273	0.81	anecdotal H0
Planning	ATL → IFC	0.26 ± 0.05	0.39 ± 0.04	−2.18	−0.56	0.033	0.150	1.86	Anecdotal H1
Planning	IC → IFC	0.48 ± 0.06	0.44 ± 0.04	0.52	0.13	0.606	0.682	0.29	Moderate H0
Standby	IC → ATL	0.51 ± 0.05	0.39 ± 0.06	1.54	0.40	0.129	0.289	0.71	Anecdotal H0
Standby	IFC → ATL	0.41 ± 0.05	0.39 ± 0.05	0.30	0.08	0.768	0.768	0.27	Moderate H0
Standby	ATL → IC	0.40 ± 0.05	0.51 ± 0.06	−1.44	−0.37	0.155	0.311	0.62	Anecdotal H0
Standby	IFC → IC	0.44 ± 0.05	0.37 ± 0.06	0.89	0.23	0.376	0.521	0.37	Anecdotal H0
Standby	ATL → IFC	0.38 ± 0.06	0.44 ± 0.05	−0.71	−0.18	0.480	0.617	0.32	Moderate H0
Standby	IC → IFC	0.52 ± 0.06	0.46 ± 0.07	0.56	0.14	0.578	0.682	0.30	Moderate H0
Perception	IC → ATL	0.54 ± 0.05	0.46 ± 0.06	1.09	0.28	0.281	0.422	0.43	Anecdotal H0
Perception	IFC → ATL	0.54 ± 0.05	0.36 ± 0.05	2.49	0.64	0.016	0.132	3.31	Moderate H1
Perception	ATL → IC	0.33 ± 0.05	0.51 ± 0.05	−2.47	−0.64	0.017	0.132	3.17	Moderate H1
Perception	IFC → IC	0.50 ± 0.05	0.38 ± 0.05	1.64	0.42	0.106	0.273	0.80	Anecdotal H0
Perception	ATL → IFC	0.30 ± 0.04	0.42 ± 0.05	−1.74	−0.45	0.088	0.273	0.92	Anecdotal H0
Perception	IC → IFC	0.55 ± 0.06	0.45 ± 0.06	1.27	0.33	0.209	0.342	0.52	Anecdotal H0

*Note:* Mean and SEM connectivities are given for the adult and adolescent groups. Also given are *t* value (*t*), Cohens's *d* (*d*), *p* value (*p*) and FDR corrected *p* value (*p* (FDR)). Significant *p* values after FDR correction are marked with an asterisk. The Bayes factor strength of evidence label is given as evidence towards H1 if BF10 > 1 and otherwise as evidence towards H0 based on Lee and Wagenmakers (2014).

## 4 Discussion

The current study asked how neurophysiological processes underlying AEB processes important for the emergence of intentional actions develop from adolescence to adulthood. To this end, we examined AEB in an established paradigm being able to dissociate between different phases to acquire AEB (i.e., the action planning phase, the anticipation phase and the perception phase) (Mayer, Helin Koyun, et al. 2025; Mayer, Mückschel, et al. 2025). In particular, we were interested in the directed network communication within theta band activity and its differences between adolescence and adulthood. Within the adolescent group, similar to the previously published adult group, we observed robust modulations of theta band activity. For adults, it has been argued (Mayer, Helin Koyun, et al. 2025; Mayer, Mückschel, et al. 2025) that the ATL receives sensorimotor data from the IC, processes this information to form a cohesive representation, and then sends feedback to the IC. In this way, the ATL and IC likely create a feedback loop for integrating information related to action planning and perception of action effects. The ATL seems to play a central role in these processes. It is plausible that the IC transmits sensorimotor signals to the ATL, which is thought to synthesize coherent concepts from diverse sensory inputs. Based on this function, the IC might supply the integrated event file that contains (or represents) the action effect (Mayer, Helin Koyun, et al. 2025; Mayer, Mückschel, et al. 2025). Notably, the adolescent group revealed a cortical network that largely resembled the one observed in adults (Mayer, Helin Koyun, et al. 2025; Mayer, Mückschel, et al. 2025). Adolescents also show an involvement of the insular cortex (IC), the anterior temporal lobe (ATL) and the inferior frontal cortex (IFC) (cf. Figure 5). This was the case for all phases of the experiment (i.e., the action planning phase, the anticipation phase and the perception phase). As found in adults, all involved brain structures revealed a directed information transfer in adolescent participants as evidenced by surrogate testing of the identified network communication using nCREANN (cf. Figures 6 and 7). The IC‐ATL‐IFC network thus likely reflects a canonical network involved in AEB processes and as such probably a canonical network for intentional actions. The finding that the IC‐ATL‐IFC network was evident in adolescents suggests that it reflects a network that is mature early on in cognitive development. All involved brain areas are likely to contribute fundamental computational principles for action‐effect integration (Binney et al. 2012; Droutman et al. 2015; Gogolla 2017; Rice et al. 2015). This may explain why the IC‐ATL‐IFC network is already involved in action‐effect integration during adolescence.

In addition to this shared core network, our data revealed notable anatomical differences in cortical architecture between adolescents and adults. Most prominently, a cluster in the lingual gyrus (LG)—part of the posterior ventral visual stream—was specifically evident in the adolescent group. This region emerged from a data‐driven voxel selection and clustering procedure applied identically to both groups, selecting the top 3% of theta‐band active voxels per phase. No comparable LG activation was observed in adults, suggesting a developmentally specific recruitment of posterior perceptual regions during AEB. While we did not perform formal voxel‐wise comparisons due to the inherent spatial limitations of EEG source reconstruction, the data‐driven procedure suggests that the divergence in functional ROI composition between groups is systematic and anatomically interpretable. These differences likely reflect maturational changes in posterior brain regions, including the ventral visual stream (Mills et al. 2014; Walhovd et al. 2015). They suggest that adolescents rely on broader, perceptually anchored networks to support action‐effect integration during development.

Yet, the involvement of the IC‐ATL‐IFC network and directed communication therein in adolescence does not imply that the organization of directed communication shows no intricacies in adolescence. This is evidenced by the comparison of the directed connectivity strength between the IC, the ATL, and the IFC between adolescent and adult participants (cf. Table 3). Group differences were evident in all phases of the experiment (i.e., the action planning phase, the anticipation phase and the perception phase). For all connections, it is shown that the directed connectivity strengths were stronger for adolescents than adults, suggesting that action‐effect integration requires stronger directed information transfer. It is possible that this reflects a necessity to compensate for presumably immature structural connectivities in adolescence. More important, however, is finding that out of all directed connections compared using FDR correction, a pattern appears in which especially the ATL was evident in the vast majority of connections revealing differences between adolescents and adults. Out of the *N* = 7 directed connectivities shown to differ between adolescent and adult participants (i.e., that withstood FDR‐correction), all involved the ATL, *N* = 4 involved the IC, and *N* = 3 involved the IFC (cf. Table 3).

To ensure clarity in interpreting the connectivity results, we emphasize the distinction between exploratory and statistically robust findings. While several directional differences in connectivity were observed between adolescents and adults across various phases of the task, only a subset of these differences remained significant after applying FDR correction. Specifically, seven directed connectivities survived correction, all of which involved the ATL, highlighting its central role in developmental differences in AEB‐related network dynamics (cf. Table 3). These findings are interpreted as statistically robust. Other directional effects reported in the results—particularly those not surviving FDR correction—should be interpreted with caution. We report these patterns to inform future hypotheses and emphasize transparency in distinguishing between preliminary trends and reliable group differences.

Thus, especially the ATL and its directed communication to the other parts of the ATL‐IC‐IFC network is differentially modulated in adolescents. As mentioned, the ATL likely acts as a representational hub (Binney et al. 2016; Hoffman et al. 2015) whose function is to distill coherent concepts from multimodal inputs (Rice et al. 2015) and the right ATL is specialized for visual processes (Rice et al. 2015). As shown in Table 3, especially the impact of the ATL on the IC was different in all phases of the AEB experiment. The role of the ATL‐IC connection must therefore be independent of the incoming information. Therefore, it is possible that the ATL provides a representational template for how multimodal information is processed in the IC (Droutman et al. 2015; Gogolla 2017). Concerning the directed connectivities with the IFC, the ATL differentially impacts the IFC in the action planning and anticipation phases but not in the perception phase (differential impact = adolescent vs. adult difference). This suggests a possible adaptive mechanism whereby the ATL helps to shape the way information is processed depending on the phase of the experiment that the ATL, and, again, corroborates that the ATL may function as a template or guide during action‐effect integration processes. It is this guidance that is differentially affected in adolescence and adulthood; i.e., it is stronger in adolescents than adult participants, as indicated by the higher values of the degree of information transfer provided by nCREANN. Selectively for the action planning phase, however, the impact of the IFC on the ATL was also different between the groups. Within the action planning phase, predictions of the precise effect it produces must be computed, and this is known to depend on theta band activity (Hovsepyan et al. 2020; Jamous et al. 2024; Sauseng et al. 2015). The (right) IFC is involved in attentional control functions (Hampshire et al. 2010), which also operate through theta band activity (Wendiggensen et al. 2022). It is possible that this supports the function of the ATL to provide templates. Taken together, adolescents rely more heavily on ATL‐driven guidance for processing multimodal information during action‐effect integration, especially in phases like planning and anticipation. The increased connectivity in adolescents indicates that these processes are not yet as automatic or mature as in adults.

Interestingly, in adolescents' theta band activity was not confined to the IC‐ATL‐IFC also observed in adults. Rather, additional theta band activity in posterior parts of the ventral visual stream (i.e., the lingual gyrus, LG) was evident in all phases of the experiment (cf. Figure 5). This region was identified through a data‐driven clustering approach that selected the top 3% of voxels contributing most strongly to theta activity, independent of predefined anatomical constraints. Among these, a coherent and anatomically plausible cluster emerged in the LG for adolescents, but not for adults, suggesting a developmentally specific pattern of engagement. Especially in the action planning and perception phases of the AEB‐experiment, the ATL had a stronger impact on the LG than vice versa. The ventral stream is involved in the processing of the identity of incoming sensory information (i.e., “what” is presented) (Goodale et al. 2005). Ideomotor principles propose that acting involves activating the neural representation of the intended action‐effect (Hommel et al. 2001; Shin et al. 2010; Waszak et al. 2012). The action's success is evaluated by comparing actual sensory effects with expected outcomes (Waszak et al. 2012). In the used AEB‐experiment, the action‐effect was a visual one and involved two distinct stimuli (i.e., picture of cat or a sheep). The impact of the ATL in the action planning phase could be interpreted as preparational activity for the action‐effect and in the perception phase as an indicator of the verification of which action‐effect was obtained. This pattern fits with the above interpretation of the role of the ATL to likely reflect a template important to guide action‐effect integration. The finding that an ATL effect on ventral stream areas was only seen in the adolescent group indicates that the action‐effect verification process as an element of ideomotor principles is supposedly more demanding to accomplish than in adults.

This interpretation is further supported when viewed through the lens of broader developmental and ideomotor frameworks outlined in the introduction. While the basic mechanisms of AEB are present from early infancy, their flexible and goal‐directed use matures over time—likely in parallel with neurodevelopmental changes in frontal and cingulate systems. For instance, reduced performance monitoring and ongoing maturation of the anterior cingulate cortex (ACC) during adolescence (Hogan et al. 2005; Ladouceur et al. 2007) may contribute to the stronger and more distributed connectivity we observed in adolescents, particularly in prefrontal and posterior regions. This enhanced engagement could serve as a compensatory mechanism to support action control under still‐developing internal monitoring systems.

At the same time, the pattern of theta‐band connectivity—especially involving the ATL and ventral stream—aligns well with ideomotor theory, which posits that actions are guided by internal representations of anticipated sensory outcomes (Hommel et al. 2001; Shin et al. 2010). The increased ATL influence seen in adolescents may reflect a developmental need for stronger top‐down scaffolding when generating or verifying such internal action‐effect templates. As the system matures, this process likely becomes more efficient and neurally streamlined, supporting the transition toward more automatic goal‐directed behavior. These findings may be particularly relevant for understanding atypical development of action control in neurodevelopmental disorders such as ADHD and ASD, where impairments in goal‐directed behavior, cognitive flexibility, and sensory integration are well documented. Although the ATL is traditionally associated with semantic processing, our results suggest a broader function in integrating conceptual representations with motor planning. Disruption of this integrative ATL function could contribute to executive and behavioral difficulties observed in clinical populations. Interventions targeting ATL‐mediated processes—such as cognitive training, neuromodulation, or behavioral therapies—may offer novel pathways to improve intentional action control in these groups.

## 5 Conclusions

In summary, this study examined developmental differences in the neural mechanisms underlying AEB in adolescents and adults. Both groups showed involvement of the IC, ATL, and IFC networks, reflecting a canonical system for intentional actions, with the ATL acting as a central hub that integrates multimodal sensory data. However, adolescents exhibited stronger directed connectivity within the IC‐ATL‐IFC network compared to adults, suggesting that action‐effect integration in adolescents requires more robust communication. The ATL's role in guiding action‐effect integration was particularly pronounced in adolescents. The greater reliance on the ATL in adolescents suggests that AEB is more demanding, aligning with the developmental perspective that cognitive processes mature over time. Additionally, adolescents showed theta band activity in the posterior ventral visual stream, specifically the LG. The ATL had a stronger impact on the LG in adolescents, especially in the action planning and perception phases, suggesting that action‐effect verification is more demanding in this age group. These findings deepen our understanding of the neurodevelopmental trajectory of intentional action and point to potential targets for interventions in clinical populations with impaired executive functioning.

## Author Contributions

All authors had full access to the data, gave final approval for publication, and agreed to be held accountable for the work performed therein. **J.M**. conceptualization, investigation, methodology, formal analysis, visualization, writing – original draft. **M.M**. formal analysis, methodology, software, visualization, writing – original draft. **B.H**. funding acquisition, conceptualization, writing – review and editing. **C.B**. conceptualization, funding acquisition, methodology, resources, supervision, writing – original draft. All authors approved the final version of the manuscript.

## Ethics Statement

All participants provided written informed consent and received a financial reimbursement for their participation in the study. The ethics committee of TU Dresden approved this study.

## Consent

The authors have nothing to report.

## Conflicts of Interest

The authors declare no conflicts of interest.

## Data Availability

The data that support the findings of this study are available from the corresponding author upon reasonable request.
